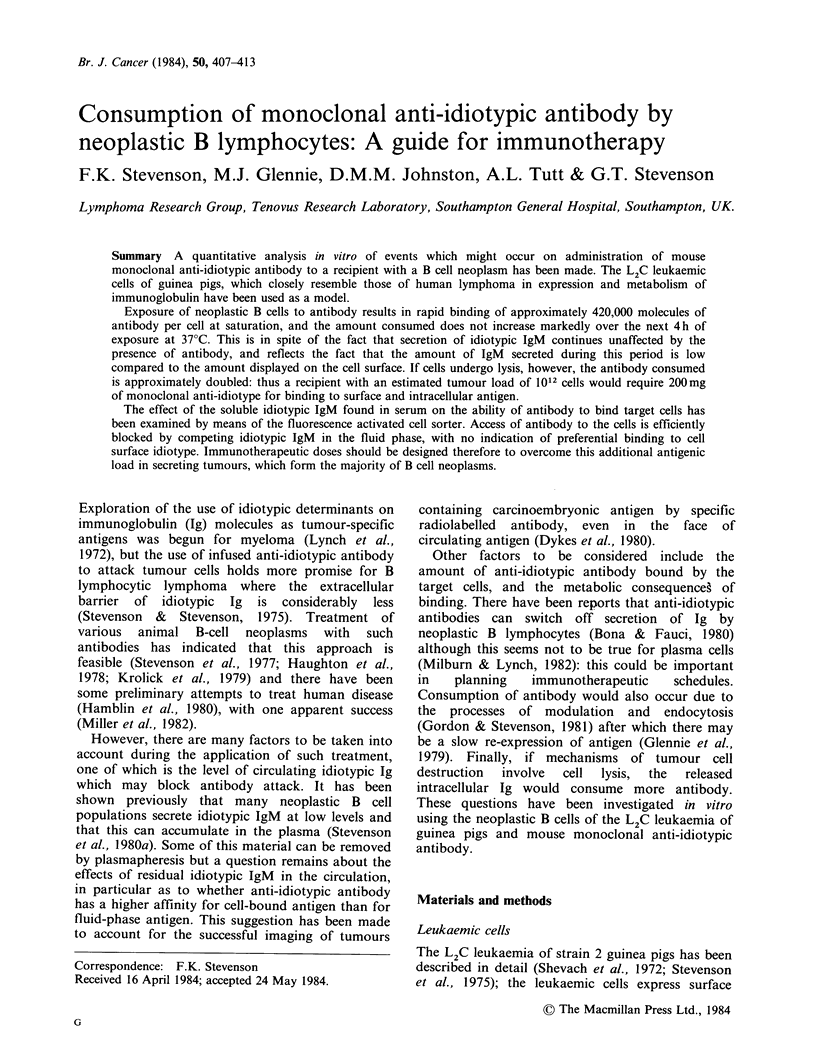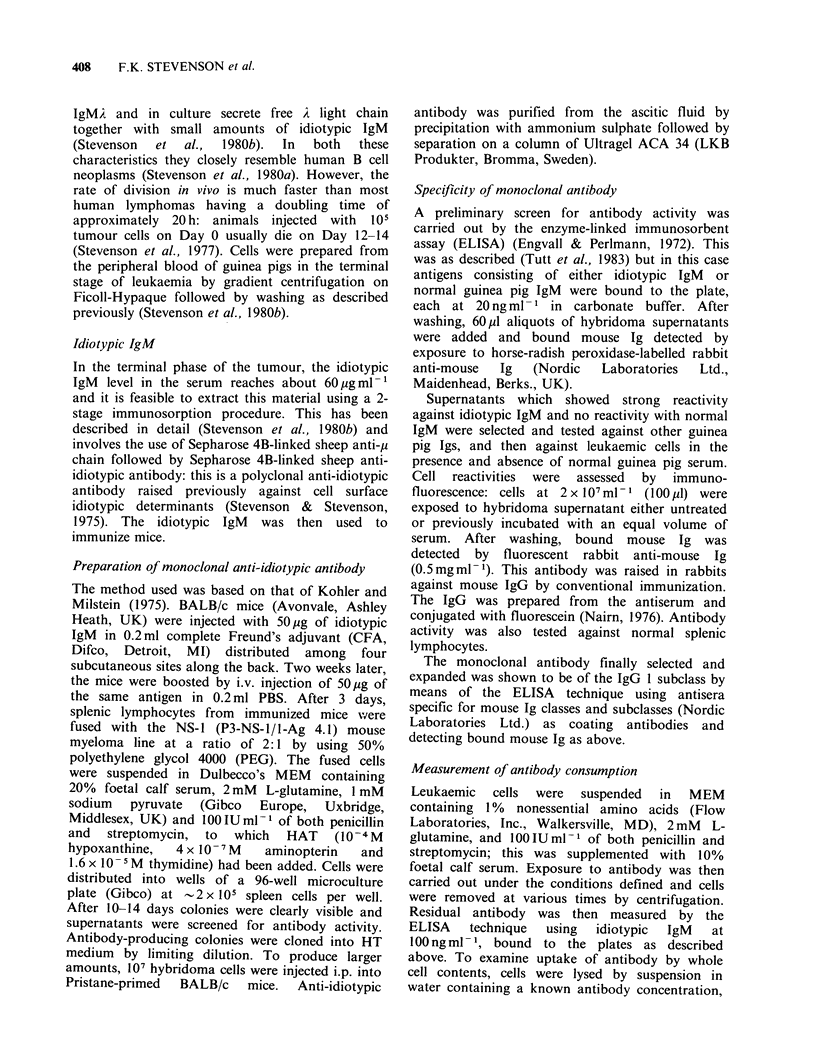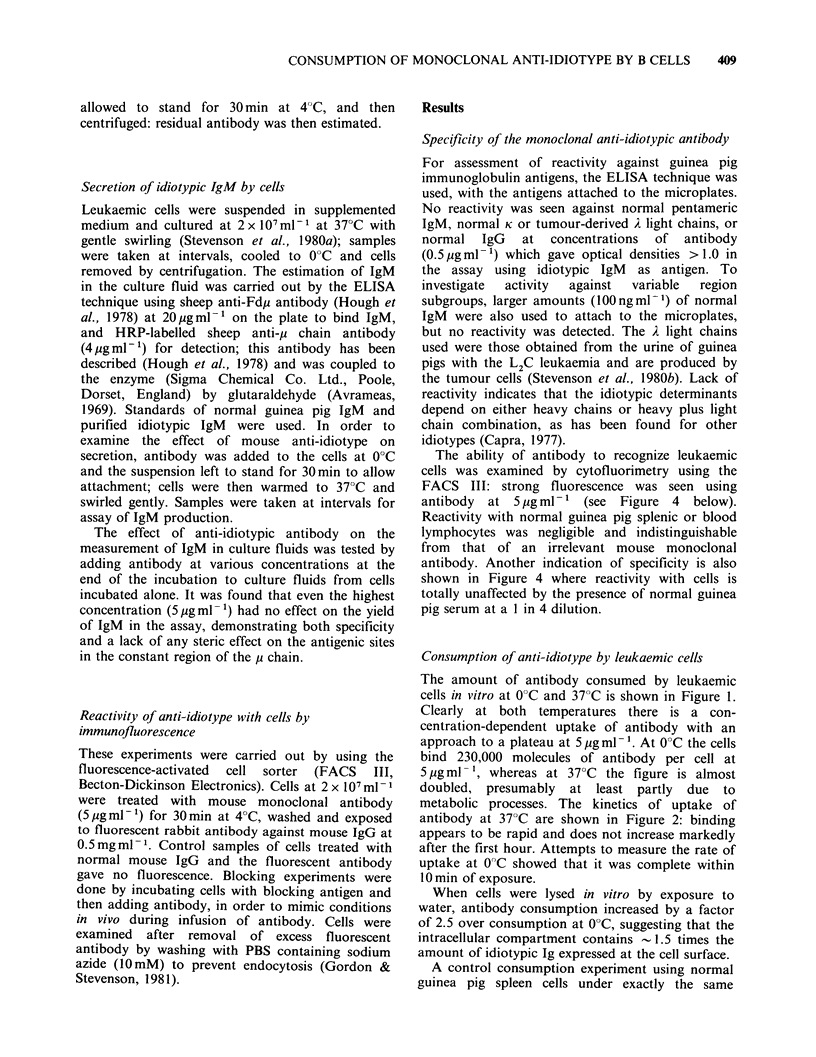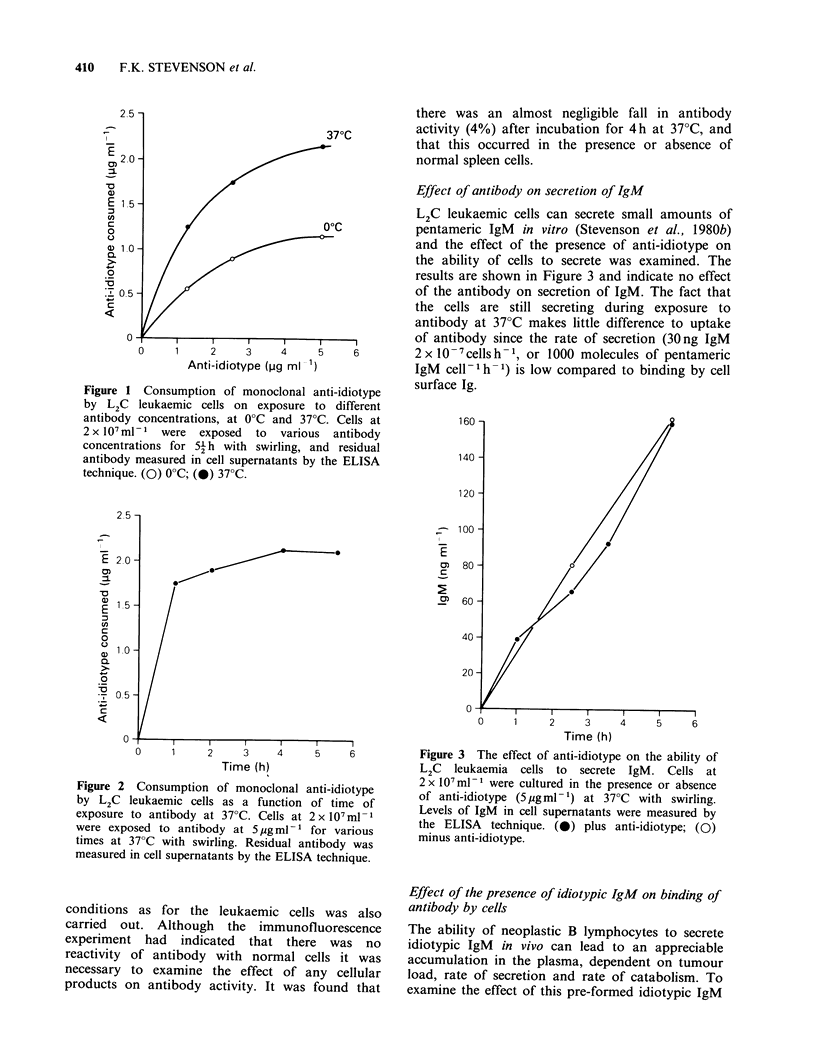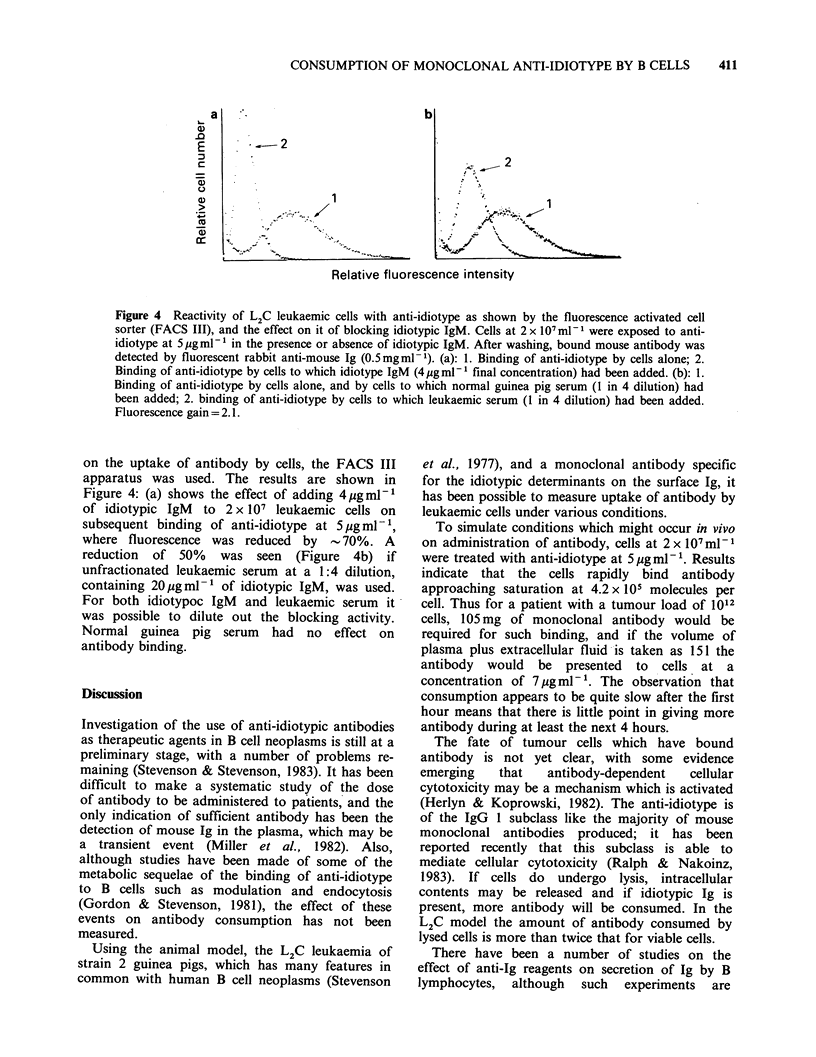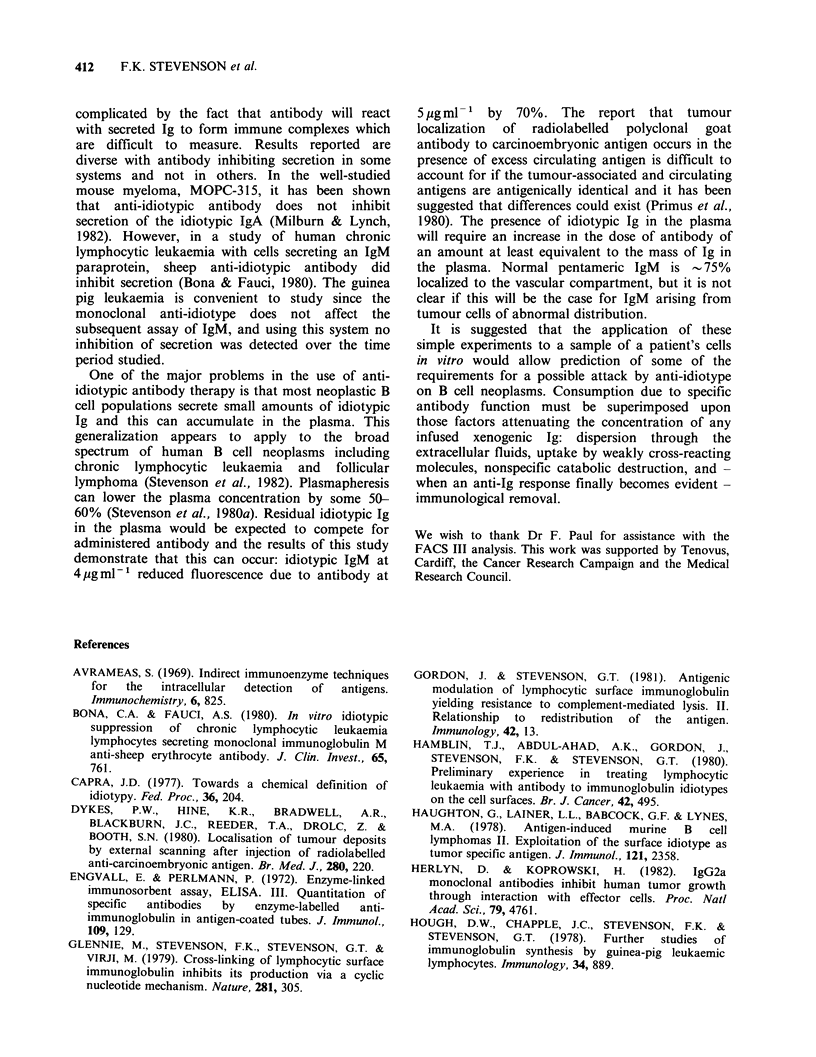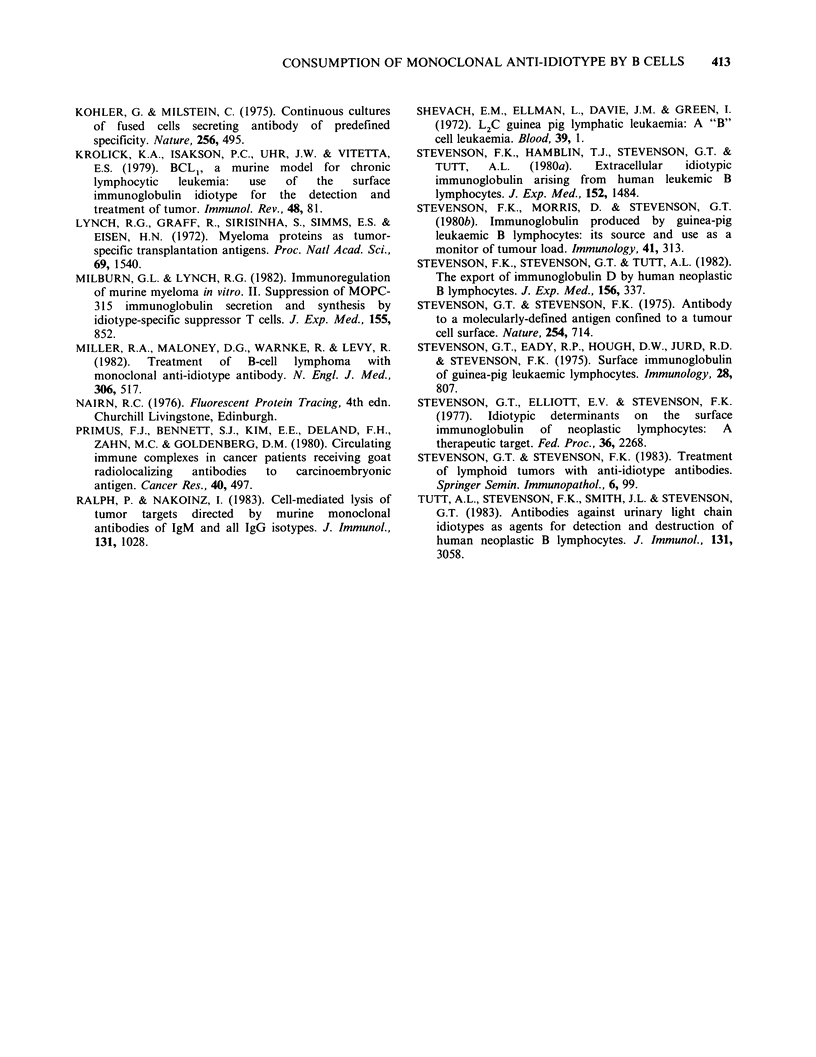# Consumption of monoclonal anti-idiotypic antibody by neoplastic B lymphocytes: a guide for immunotherapy.

**DOI:** 10.1038/bjc.1984.190

**Published:** 1984-09

**Authors:** F. K. Stevenson, M. J. Glennie, D. M. Johnston, A. L. Tutt, G. T. Stevenson

## Abstract

A quantitative analysis in vitro of events which might occur on administration of mouse monoclonal anti-idiotypic antibody to a recipient with a B cell neoplasm has been made. The L2C leukaemic cells of guinea pigs, which closely resemble those of human lymphoma in expression and metabolism of immunoglobulin have been used as a model. Exposure of neoplastic B cells to antibody results in rapid binding of approximately 420,000 molecules of antibody per cell at saturation, and the amount consumed does not increase markedly over the next 4 h of exposure at 37 degrees C. This is in spite of the fact that secretion of idiotypic IgM continues unaffected by the presence of antibody, and reflects the fact that the amount of IgM secreted during this period is low compared to the amount displayed on the cell surface. If cells undergo lysis, however, the antibody consumed is approximately doubled: thus a recipient with an estimated tumour load of 10(12) cells would require 200 mg of monoclonal anti-idiotype for binding to surface and intracellular antigen. The effect of the soluble idiotypic IgM found in serum on the ability of antibody to bind target cells has been examined by means of the fluorescence activated cell sorter. Access of antibody to the cells is efficiently blocked by competing idiotypic IgM in the fluid phase, with no indication of preferential binding to cell surface idiotype. Immunotherapeutic doses should be designed therefore to overcome this additional antigenic load in secreting tumours, which form the majority of B cell neoplasms.


*Br. J. Cancer* (1984), **50**, 407–413

### Consumption of monoclonal anti-idiotypic antibody by neoplastic B lymphocytes: A guide for immunotherapy

F.K. Stevenson, M.J. Glennie, D.M.M. Johnston, A.L. Tutt & G.T. Stevenson

*Lymphoma Research Group, Tenovus Research Laboratory, Southampton General Hospital, Southampton, UK.*

**Summary** A quantitative analysis *in vitro* of events which might occur on administration of mouse monoclonal anti-idiotypic antibody to a recipient with a B cell neoplasm has been made. The $L_2C$ leukaemic cells of guinea pigs, which closely resemble those of human lymphoma in expression and metabolism of immunoglobulin have been used as a model.

Exposure of neoplastic B cells to antibody results in rapid binding of approximately 420,000 molecules of antibody per cell at saturation, and the amount consumed does not increase markedly over the next 4 h of exposure at 37°C. This is in spite of the fact that secretion of idiotypic IgM continues unaffected by the presence of antibody, and reflects the fact that the amount of IgM secreted during this period is low compared to the amount displayed on the cell surface. If cells undergo lysis, however, the antibody consumed is approximately doubled: thus a recipient with an estimated tumour load of $10^{12}$ cells would require 200 mg of monoclonal anti-idiotype for binding to surface and intracellular antigen.

The effect of the soluble idiotypic IgM found in serum on the ability of antibody to bind target cells has been examined by means of the fluorescence activated cell sorter. Access of antibody to the cells is efficiently blocked by competing idiotypic IgM in the fluid phase, with no indication of preferential binding to cell surface idiotype. Immunotherapeutic doses should be designed therefore to overcome this additional antigenic load in secreting tumours, which form the majority of B cell neoplasms.

Exploration of the use of idiotypic determinants on immunoglobulin (Ig) molecules as tumour-specific antigens was begun for myeloma (Lynch *et al.*, 1972), but the use of infused anti-idiotypic antibody to attack tumour cells holds more promise for B lymphocytic lymphoma where the extracellular barrier of idiotypic Ig is considerably less (Stevenson & Stevenson, 1975). Treatment of various animal B-cell neoplasms with such antibodies has indicated that this approach is feasible (Stevenson *et al.*, 1977; Haughton *et al.*, 1978; Krolick *et al.*, 1979) and there have been some preliminary attempts to treat human disease (Hamblin *et al.*, 1980), with one apparent success (Miller *et al.*, 1982).

However, there are many factors to be taken into account during the application of such treatment, one of which is the level of circulating idiotypic Ig which may block antibody attack. It has been shown previously that many neoplastic B cell populations secrete idiotypic IgM at low levels and that this can accumulate in the plasma (Stevenson *et al.*, 1980*a*). Some of this material can be removed by plasmapheresis but a question remains about the effects of residual idiotypic IgM in the circulation, in particular as to whether anti-idiotypic antibody has a higher affinity for cell-bound antigen than for fluid-phase antigen. This suggestion has been made to account for the successful imaging of tumours containing carcinoembryonic antigen by specific radiolabelled antibody, even in the face of circulating antigen (Dykes *et al.*, 1980).

Other factors to be considered include the amount of anti-idiotypic antibody bound by the target cells, and the metabolic consequences of binding. There have been reports that anti-idiotypic antibodies can switch off secretion of Ig by neoplastic B lymphocytes (Bona & Fauci, 1980) although this seems not to be true for plasma cells (Milburn & Lynch, 1982): this could be important in planning immunotherapeutic schedules. Consumption of antibody would also occur due to the processes of modulation and endocytosis (Gordon & Stevenson, 1981) after which there may be a slow re-expression of antigen (Glennie *et al.*, 1979). Finally, if mechanisms of tumour cell destruction involve cell lysis, the released intracellular Ig would consume more antibody. These questions have been investigated *in vitro* using the neoplastic B cells of the $L_2C$ leukaemia of guinea pigs and mouse monoclonal anti-idiotypic antibody.

### Materials and methods

### *Leukaemic cells*

The $L_2C$ leukaemia of strain 2 guinea pigs has been described in detail (Shevach *et al.*, 1972; Stevenson *et al.*, 1975); the leukaemic cells express surface

Correspondence: F.K. Stevenson
Received 16 April 1984; accepted 24 May 1984.

© The Macmillan Press Ltd., 1984

IgMλ and in culture secrete free λ light chain together with small amounts of idiotypic IgM (Stevenson *et al.*, 1980*b*). In both these characteristics they closely resemble human B cell neoplasms (Stevenson *et al.*, 1980*a*). However, the rate of division *in vivo* is much faster than most human lymphomas having a doubling time of approximately 20 h: animals injected with $10^5$ tumour cells on Day 0 usually die on Day 12–14 (Stevenson *et al.*, 1977). Cells were prepared from the peripheral blood of guinea pigs in the terminal stage of leukaemia by gradient centrifugation on Ficoll-Hypaque followed by washing as described previously (Stevenson *et al.*, 1980*b*).

*Idiotypic IgM*

In the terminal phase of the tumour, the idiotypic IgM level in the serum reaches about $60 \mu g ml^{-1}$ and it is feasible to extract this material using a 2-stage immunosorption procedure. This has been described in detail (Stevenson *et al.*, 1980*b*) and involves the use of Sepharose 4B-linked sheep anti-μ chain followed by Sepharose 4B-linked sheep anti-idiotypic antibody: this is a polyclonal anti-idiotypic antibody raised previously against cell surface idiotypic determinants (Stevenson & Stevenson, 1975). The idiotypic IgM was then used to immunize mice.

*Preparation of monoclonal anti-idiotypic antibody*

The method used was based on that of Kohler and Milstein (1975). BALB/c mice (Avonvale, Ashley Heath, UK) were injected with $50 \mu g$ of idiotypic IgM in 0.2 ml complete Freund's adjuvant (CFA, Difco, Detroit, MI) distributed among four subcutaneous sites along the back. Two weeks later, the mice were boosted by i.v. injection of $50 \mu g$ of the same antigen in 0.2 ml PBS. After 3 days, splenic lymphocytes from immunized mice were fused with the NS-1 (P3-NS-1/1-Ag 4.1) mouse myeloma line at a ratio of 2:1 by using 50% polyethylene glycol 4000 (PEG). The fused cells were suspended in Dulbecco's MEM containing 20% foetal calf serum, 2 mM L-glutamine, 1 mM sodium pyruvate (Gibco Europe, Uxbridge, Middlesex, UK) and $100 IU ml^{-1}$ of both penicillin and streptomycin, to which HAT ($10^{-4}$ M hypoxanthine, $4 \times 10^{-7}$ M aminopterin and $1.6 \times 10^{-5}$ M thymidine) had been added. Cells were distributed into wells of a 96-well microculture plate (Gibco) at $\sim 2 \times 10^5$ spleen cells per well. After 10–14 days colonies were clearly visible and supernatants were screened for antibody activity. Antibody-producing colonies were cloned into HT medium by limiting dilution. To produce larger amounts, $10^7$ hybridoma cells were injected i.p. into Pristane-primed BALB/c mice. Anti-idiotypic antibody was purified from the ascitic fluid by precipitation with ammonium sulphate followed by separation on a column of Ultragel ACA 34 (LKB Produkter, Bromma, Sweden).

*Specificity of monoclonal antibody*

A preliminary screen for antibody activity was carried out by the enzyme-linked immunosorbent assay (ELISA) (Engvall & Perlmann, 1972). This was as described (Tutt *et al.*, 1983) but in this case antigens consisting of either idiotypic IgM or normal guinea pig IgM were bound to the plate, each at $20 ng ml^{-1}$ in carbonate buffer. After washing, $60 \mu l$ aliquots of hybridoma supernatants were added and bound mouse Ig detected by exposure to horse-radish peroxidase-labelled rabbit anti-mouse Ig (Nordic Laboratories Ltd., Maidenhead, Berks., UK).

Supernatants which showed strong reactivity against idiotypic IgM and no reactivity with normal IgM were selected and tested against other guinea pig Igs, and then against leukaemic cells in the presence and absence of normal guinea pig serum. Cell reactivities were assessed by immunofluorescence: cells at $2 \times 10^7 ml^{-1}$ ($100 \mu l$) were exposed to hybridoma supernatant either untreated or previously incubated with an equal volume of serum. After washing, bound mouse Ig was detected by fluorescent rabbit anti-mouse Ig ($0.5 mg ml^{-1}$). This antibody was raised in rabbits against mouse IgG by conventional immunization. The IgG was prepared from the antiserum and conjugated with fluorescein (Nairn, 1976). Antibody activity was also tested against normal splenic lymphocytes.

The monoclonal antibody finally selected and expanded was shown to be of the IgG 1 subclass by means of the ELISA technique using antisera specific for mouse Ig classes and subclasses (Nordic Laboratories Ltd.) as coating antibodies and detecting bound mouse Ig as above.

*Measurement of antibody consumption*

Leukaemic cells were suspended in MEM containing 1% nonessential amino acids (Flow Laboratories, Inc., Walkersville, MD), 2 mM L-glutamine, and $100 IU ml^{-1}$ of both penicillin and streptomycin; this was supplemented with 10% foetal calf serum. Exposure to antibody was then carried out under the conditions defined and cells were removed at various times by centrifugation. Residual antibody was then measured by the ELISA technique using idiotypic IgM at $100 ng ml^{-1}$, bound to the plates as described above. To examine uptake of antibody by whole cell contents, cells were lysed by suspension in water containing a known antibody concentration, allowed to stand for 30 min at 4°C, and then centrifuged: residual antibody was then estimated.

*Secretion of idiotypic IgM by cells*

Leukaemic cells were suspended in supplemented medium and cultured at $2 \times 10^7 ml^{-1}$ at 37°C with gentle swirling (Stevenson *et al.*, 1980*a*); samples were taken at intervals, cooled to 0°C and cells removed by centrifugation. The estimation of IgM in the culture fluid was carried out by the ELISA technique using sheep anti-Fd$\mu$ antibody (Hough *et al.*, 1978) at 20 $\mu g ml^{-1}$ on the plate to bind IgM, and HRP-labelled sheep anti-$\mu$ chain antibody (4 $\mu g ml^{-1}$) for detection; this antibody has been described (Hough *et al.*, 1978) and was coupled to the enzyme (Sigma Chemical Co. Ltd., Poole, Dorset, England) by glutaraldehyde (Avrameas, 1969). Standards of normal guinea pig IgM and purified idiotypic IgM were used. In order to examine the effect of mouse anti-idiotype on secretion, antibody was added to the cells at 0°C and the suspension left to stand for 30 min to allow attachment; cells were then warmed to 37°C and swirled gently. Samples were taken at intervals for assay of IgM production.

The effect of anti-idiotypic antibody on the measurement of IgM in culture fluids was tested by adding antibody at various concentrations at the end of the incubation to culture fluids from cells incubated alone. It was found that even the highest concentration (5 $\mu g ml^{-1}$) had no effect on the yield of IgM in the assay, demonstrating both specificity and a lack of any steric effect on the antigenic sites in the constant region of the $\mu$ chain.

*Reactivity of anti-idiotype with cells by immunofluorescence*

These experiments were carried out by using the fluorescence-activated cell sorter (FACS III, Becton-Dickinson Electronics). Cells at $2 \times 10^7 ml^{-1}$ were treated with mouse monoclonal antibody (5 $\mu g ml^{-1}$) for 30 min at 4°C, washed and exposed to fluorescent rabbit antibody against mouse IgG at 0.5 $mg ml^{-1}$. Control samples of cells treated with normal mouse IgG and the fluorescent antibody gave no fluorescence. Blocking experiments were done by incubating cells with blocking antigen and then adding antibody, in order to mimic conditions *in vivo* during infusion of antibody. Cells were examined after removal of excess fluorescent antibody by washing with PBS containing sodium azide (10 mM) to prevent endocytosis (Gordon & Stevenson, 1981).

### Results

*Specificity of the monoclonal anti-idiotypic antibody*

For assessment of reactivity against guinea pig immunoglobulin antigens, the ELISA technique was used, with the antigens attached to the microplates. No reactivity was seen against normal pentameric IgM, normal $\kappa$ or tumour-derived $\lambda$ light chains, or normal IgG at concentrations of antibody (0.5 $\mu g ml^{-1}$) which gave optical densities >1.0 in the assay using idiotypic IgM as antigen. To investigate activity against variable region subgroups, larger amounts (100 $ng ml^{-1}$) of normal IgM were also used to attach to the microplates, but no reactivity was detected. The $\lambda$ light chains used were those obtained from the urine of guinea pigs with the $L_2C$ leukaemia and are produced by the tumour cells (Stevenson *et al.*, 1980*b*). Lack of reactivity indicates that the idiotypic determinants depend on either heavy chains or heavy plus light chain combination, as has been found for other idiotypes (Capra, 1977).

The ability of antibody to recognize leukaemic cells was examined by cytofluorimetry using the FACS III: strong fluorescence was seen using antibody at 5 $\mu g ml^{-1}$ (see Figure 4 below). Reactivity with normal guinea pig splenic or blood lymphocytes was negligible and indistinguishable from that of an irrelevant mouse monoclonal antibody. Another indication of specificity is also shown in Figure 4 where reactivity with cells is totally unaffected by the presence of normal guinea pig serum at a 1 in 4 dilution.

*Consumption of anti-idiotype by leukaemic cells*

The amount of antibody consumed by leukaemic cells *in vitro* at 0°C and 37°C is shown in Figure 1. Clearly at both temperatures there is a concentration-dependent uptake of antibody with an approach to a plateau at 5 $\mu g ml^{-1}$. At 0°C the cells bind 230,000 molecules of antibody per cell at 5 $\mu g ml^{-1}$, whereas at 37°C the figure is almost doubled, presumably at least partly due to metabolic processes. The kinetics of uptake of antibody at 37°C are shown in Figure 2: binding appears to be rapid and does not increase markedly after the first hour. Attempts to measure the rate of uptake at 0°C showed that it was complete within 10 min of exposure.

When cells were lysed *in vitro* by exposure to water, antibody consumption increased by a factor of 2.5 over consumption at 0°C, suggesting that the intracellular compartment contains ~1.5 times the amount of idiotypic Ig expressed at the cell surface.

A control consumption experiment using normal guinea pig spleen cells under exactly the same

**Figure 1** Consumption of monoclonal anti-idiotype by $L_2C$ leukaemic cells on exposure to different antibody concentrations, at 0°C and 37°C. Cells at $2 \times 10^7 ml^{-1}$ were exposed to various antibody concentrations for $5\frac{1}{2}$h with swirling, and residual antibody measured in cell supernatants by the ELISA technique. (○) 0°C; (●) 37°C.

**Figure 2** Consumption of monoclonal anti-idiotype by $L_2C$ leukaemic cells as a function of time of exposure to antibody at 37°C. Cells at $2 \times 10^7 ml^{-1}$ were exposed to antibody at $5 \mu g ml^{-1}$ for various times at 37°C with swirling. Residual antibody was measured in cell supernatants by the ELISA technique.

conditions as for the leukaemic cells was also carried out. Although the immunofluorescence experiment had indicated that there was no reactivity of antibody with normal cells it was necessary to examine the effect of any cellular products on antibody activity. It was found that there was an almost negligible fall in antibody activity (4%) after incubation for 4h at 37°C, and that this occurred in the presence or absence of normal spleen cells.

*Effect of antibody on secretion of IgM*

$L_2C$ leukaemic cells can secrete small amounts of pentameric IgM *in vitro* (Stevenson *et al.*, 1980*b*) and the effect of the presence of anti-idiotype on the ability of cells to secrete was examined. The results are shown in Figure 3 and indicate no effect of the antibody on secretion of IgM. The fact that the cells are still secreting during exposure to antibody at 37°C makes little difference to uptake of antibody since the rate of secretion (30 ng IgM $2 \times 10^{-7}$ cells $h^{-1}$, or 1000 molecules of pentameric IgM $cell^{-1} h^{-1}$) is low compared to binding by cell surface Ig.

**Figure 3** The effect of anti-idiotype on the ability of $L_2C$ leukaemia cells to secrete IgM. Cells at $2 \times 10^7 ml^{-1}$ were cultured in the presence or absence of anti-idiotype ($5 \mu g ml^{-1}$) at 37°C with swirling. Levels of IgM in cell supernatants were measured by the ELISA technique. (●) plus anti-idiotype; (○) minus anti-idiotype.

*Effect of the presence of idiotypic IgM on binding of antibody by cells*

The ability of neoplastic B lymphocytes to secrete idiotypic IgM *in vivo* can lead to an appreciable accumulation in the plasma, dependent on tumour load, rate of secretion and rate of catabolism. To examine the effect of this pre-formed idiotypic IgM

**Figure 4** Reactivity of $L_2C$ leukaemic cells with anti-idiotype as shown by the fluorescence activated cell sorter (FACS III), and the effect on it of blocking idiotypic IgM. Cells at $2 \times 10^7 ml^{-1}$ were exposed to anti-idiotype at $5 \mu g ml^{-1}$ in the presence or absence of idiotypic IgM. After washing, bound mouse antibody was detected by fluorescent rabbit anti-mouse Ig ($0.5 mg ml^{-1}$). (a): 1. Binding of anti-idiotype by cells alone; 2. Binding of anti-idiotype by cells to which idiotype IgM ($4 \mu g ml^{-1}$ final concentration) had been added. (b): 1. Binding of anti-idiotype by cells alone, and by cells to which normal guinea pig serum (1 in 4 dilution) had been added; 2. binding of anti-idiotype by cells to which leukaemic serum (1 in 4 dilution) had been added. Fluorescence gain = 2.1.

on the uptake of antibody by cells, the FACS III apparatus was used. The results are shown in Figure 4: (a) shows the effect of adding $4 \mu g ml^{-1}$ of idiotypic IgM to $2 \times 10^7$ leukaemic cells on subsequent binding of anti-idiotype at $5 \mu g ml^{-1}$, where fluorescence was reduced by ~70%. A reduction of 50% was seen (Figure 4b) if unfractionated leukaemic serum at a 1:4 dilution, containing $20 \mu g ml^{-1}$ of idiotypic IgM, was used. For both idiotypoc IgM and leukaemic serum it was possible to dilute out the blocking activity. Normal guinea pig serum had no effect on antibody binding.

### Discussion

Investigation of the use of anti-idiotypic antibodies as therapeutic agents in B cell neoplasms is still at a preliminary stage, with a number of problems remaining (Stevenson & Stevenson, 1983). It has been difficult to make a systematic study of the dose of antibody to be administered to patients, and the only indication of sufficient antibody has been the detection of mouse Ig in the plasma, which may be a transient event (Miller *et al.*, 1982). Also, although studies have been made of some of the metabolic sequelae of the binding of anti-idiotype to B cells such as modulation and endocytosis (Gordon & Stevenson, 1981), the effect of these events on antibody consumption has not been measured.

Using the animal model, the $L_2C$ leukaemia of strain 2 guinea pigs, which has many features in common with human B cell neoplasms (Stevenson *et al.*, 1977), and a monoclonal antibody specific for the idiotypic determinants on the surface Ig, it has been possible to measure uptake of antibody by leukaemic cells under various conditions.

To simulate conditions which might occur *in vivo* on administration of antibody, cells at $2 \times 10^7 ml^{-1}$ were treated with anti-idiotype at $5 \mu g ml^{-1}$. Results indicate that the cells rapidly bind antibody approaching saturation at $4.2 \times 10^5$ molecules per cell. Thus for a patient with a tumour load of $10^{12}$ cells, 105 mg of monoclonal antibody would be required for such binding, and if the volume of plasma plus extracellular fluid is taken as 15 l the antibody would be presented to cells at a concentration of $7 \mu g ml^{-1}$. The observation that consumption appears to be quite slow after the first hour means that there is little point in giving more antibody during at least the next 4 hours.

The fate of tumour cells which have bound antibody is not yet clear, with some evidence emerging that antibody-dependent cellular cytotoxicity may be a mechanism which is activated (Herlyn & Koprowski, 1982). The anti-idiotype is of the IgG 1 subclass like the majority of mouse monoclonal antibodies produced; it has been reported recently that this subclass is able to mediate cellular cytotoxicity (Ralph & Nakoinz, 1983). If cells do undergo lysis, intracellular contents may be released and if idiotypic Ig is present, more antibody will be consumed. In the $L_2C$ model the amount of antibody consumed by lysed cells is more than twice that for viable cells.

There have been a number of studies on the effect of anti-Ig reagents on secretion of Ig by B lymphocytes, although such experiments are complicated by the fact that antibody will react with secreted Ig to form immune complexes which are difficult to measure. Results reported are diverse with antibody inhibiting secretion in some systems and not in others. In the well-studied mouse myeloma, MOPC-315, it has been shown that anti-idiotypic antibody does not inhibit secretion of the idiotypic IgA (Milburn & Lynch, 1982). However, in a study of human chronic lymphocytic leukaemia with cells secreting an IgM paraprotein, sheep anti-idiotypic antibody did inhibit secretion (Bona & Fauci, 1980). The guinea pig leukaemia is convenient to study since the monoclonal anti-idiotype does not affect the subsequent assay of IgM, and using this system no inhibition of secretion was detected over the time period studied.

One of the major problems in the use of anti-idiotypic antibody therapy is that most neoplastic B cell populations secrete small amounts of idiotypic Ig and this can accumulate in the plasma. This generalization appears to apply to the broad spectrum of human B cell neoplasms including chronic lymphocytic leukaemia and follicular lymphoma (Stevenson *et al.*, 1982). Plasmapheresis can lower the plasma concentration by some 50–60% (Stevenson *et al.*, 1980*a*). Residual idiotypic Ig in the plasma would be expected to compete for administered antibody and the results of this study demonstrate that this can occur: idiotypic IgM at $4 \mu g\, ml^{-1}$ reduced fluorescence due to antibody at $5 \mu g\, ml^{-1}$ by 70%. The report that tumour localization of radiolabelled polyclonal goat antibody to carcinoembryonic antigen occurs in the presence of excess circulating antigen is difficult to account for if the tumour-associated and circulating antigens are antigenically identical and it has been suggested that differences could exist (Primus *et al.*, 1980). The presence of idiotypic Ig in the plasma will require an increase in the dose of antibody of an amount at least equivalent to the mass of Ig in the plasma. Normal pentameric IgM is ~75% localized to the vascular compartment, but it is not clear if this will be the case for IgM arising from tumour cells of abnormal distribution.

It is suggested that the application of these simple experiments to a sample of a patient's cells *in vitro* would allow prediction of some of the requirements for a possible attack by anti-idiotype on B cell neoplasms. Consumption due to specific antibody function must be superimposed upon those factors attenuating the concentration of any infused xenogenic Ig: dispersion through the extracellular fluids, uptake by weakly cross-reacting molecules, nonspecific catabolic destruction, and – when an anti-Ig response finally becomes evident – immunological removal.

We wish to thank Dr F. Paul for assistance with the FACS III analysis. This work was supported by Tenovus, Cardiff, the Cancer Research Campaign and the Medical Research Council.

### References

AVRAMEAS, S. (1969). Indirect immunoenzyme techniques for the intracellular detection of antigens. *Immunochemistry*, **6**, 825.

BONA, C.A. & FAUCI, A.S. (1980). *In vitro* idiotypic suppression of chronic lymphocytic leukaemia lymphocytes secreting monoclonal immunoglobulin M anti-sheep erythrocyte antibody. *J. Clin. Invest.*, **65**, 761.

CAPRA, J.D. (1977). Towards a chemical definition of idiotypy. *Fed. Proc.*, **36**, 204.

DYKES, P.W., HINE, K.R., BRADWELL, A.R., BLACKBURN, J.C., REEDER, T.A., DROLC, Z. & BOOTH, S.N. (1980). Localisation of tumour deposits by external scanning after injection of radiolabelled anti-carcinoembryonic antigen. *Br. Med. J.*, **280**, 220.

ENGVALL, E. & PERLMANN, P. (1972). Enzyme-linked immunosorbent assay, ELISA. III. Quantitation of specific antibodies by enzyme-labelled anti-immunoglobulin in antigen-coated tubes. *J. Immunol.*, **109**, 129.

GLENNIE, M., STEVENSON, F.K., STEVENSON, G.T. & VIRJI, M. (1979). Cross-linking of lymphocytic surface immunoglobulin inhibits its production via a cyclic nucleotide mechanism. *Nature*, **281**, 305.

GORDON, J. & STEVENSON, G.T. (1981). Antigenic modulation of lymphocytic surface immunoglobulin yielding resistance to complement-mediated lysis. II. Relationship to redistribution of the antigen. *Immunology*, **42**, 13.

HAMBLIN, T.J., ABDUL-AHAD, A.K., GORDON, J., STEVENSON, F.K. & STEVENSON, G.T. (1980). Preliminary experience in treating lymphocytic leukaemia with antibody to immunoglobulin idiotypes on the cell surfaces. *Br. J. Cancer*, **42**, 495.

HAUGHTON, G., LAINER, L.L., BABCOCK, G.F. & LYNES, M.A. (1978). Antigen-induced murine B cell lymphomas II. Exploitation of the surface idiotype as tumor specific antigen. *J. Immunol.*, **121**, 2358.

HERLYN, D. & KOPROWSKI, H. (1982). IgG2a monoclonal antibodies inhibit human tumor growth through interaction with effector cells. *Proc. Natl Acad. Sci.*, **79**, 4761.

HOUGH, D.W., CHAPPLE, J.C., STEVENSON, F.K. & STEVENSON, G.T. (1978). Further studies of immunoglobulin synthesis by guinea-pig leukaemic lymphocytes. *Immunology*, **34**, 889.

KOHLER, G. & MILSTEIN, C. (1975). Continuous cultures of fused cells secreting antibody of predefined specificity. *Nature*, **256**, 495.

KROLICK, K.A., ISAKSON, P.C., UHR, J.W. & VITETTA, E.S. (1979). $BCL_1$, a murine model for chronic lymphocytic leukemia: use of the surface immunoglobulin idiotype for the detection and treatment of tumor. *Immunol. Rev.*, **48**, 81.

LYNCH, R.G., GRAFF, R., SIRISINHA, S., SIMMS, E.S. & EISEN, H.N. (1972). Myeloma proteins as tumor-specific transplantation antigens. *Proc. Natl Acad. Sci.*, **69**, 1540.

MILBURN, G.L. & LYNCH, R.G. (1982). Immunoregulation of murine myeloma *in vitro*. II. Suppression of MOPC-315 immunoglobulin secretion and synthesis by idiotype-specific suppressor T cells. *J. Exp. Med.*, **155**, 852.

MILLER, R.A., MALONEY, D.G., WARNKE, R. & LEVY, R. (1982). Treatment of B-cell lymphoma with monoclonal anti-idiotype antibody. *N. Engl. J. Med.*, **306**, 517.

NAIRN, R.C. (1976). *Fluorescent Protein Tracing*, 4th edn. Churchill Livingstone, Edinburgh.

PRIMUS, F.J., BENNETT, S.J., KIM, E.E., DELAND, F.H., ZAHN, M.C. & GOLDENBERG, D.M. (1980). Circulating immune complexes in cancer patients receiving goat radiolocalizing antibodies to carcinoembryonic antigen. *Cancer Res.*, **40**, 497.

RALPH, P. & NAKOINZ, I. (1983). Cell-mediated lysis of tumor targets directed by murine monoclonal antibodies of IgM and all IgG isotypes. *J. Immunol.*, **131**, 1028.

SHEVACH, E.M., ELLMAN, L., DAVIE, J.M. & GREEN, I. (1972). $L_2C$ guinea pig lymphatic leukaemia: A "B" cell leukaemia. *Blood*, **39**, 1.

STEVENSON, F.K., HAMBLIN, T.J., STEVENSON, G.T. & TUTT, A.L. (1980*a*). Extracellular idiotypic immunoglobulin arising from human leukemic B lymphocytes. *J. Exp. Med.*, **152**, 1484.

STEVENSON, F.K., MORRIS, D. & STEVENSON, G.T. (1980*b*). Immunoglobulin produced by guinea-pig leukaemic B lymphocytes: its source and use as a monitor of tumour load. *Immunology*, **41**, 313.

STEVENSON, F.K., STEVENSON, G.T. & TUTT, A.L. (1982). The export of immunoglobulin D by human neoplastic B lymphocytes. *J. Exp. Med.*, **156**, 337.

STEVENSON, G.T. & STEVENSON, F.K. (1975). Antibody to a molecularly-defined antigen confined to a tumour cell surface. *Nature*, **254**, 714.

STEVENSON, G.T., EADY, R.P., HOUGH, D.W., JURD, R.D. & STEVENSON, F.K. (1975). Surface immunoglobulin of guinea-pig leukaemic lymphocytes. *Immunology*, **28**, 807.

STEVENSON, G.T., ELLIOTT, E.V. & STEVENSON, F.K. (1977). Idiotypic determinants on the surface immunoglobulin of neoplastic lymphocytes: A therapeutic target. *Fed. Proc.*, **36**, 2268.

STEVENSON, G.T. & STEVENSON, F.K. (1983). Treatment of lymphoid tumors with anti-idiotype antibodies. *Springer Semin. Immunopathol.*, **6**, 99.

TUTT, A.L., STEVENSON, F.K., SMITH, J.L. & STEVENSON, G.T. (1983). Antibodies against urinary light chain idiotypes as agents for detection and destruction of human neoplastic B lymphocytes. *J. Immunol.*, **131**, 3058.